# A pharmacoinformatic approach for studying *Atractylodes Lancea* DC’s anticancer potential and control ROS-mediated apoptosis against prostate cancer cells

**DOI:** 10.3389/fonc.2025.1471110

**Published:** 2025-02-05

**Authors:** Chi-Hoon Ahn, Ji Soo Myong, Kazi Rejvee Ahmed, Md Ataur Rahman, Md. Maharub Hossain Fahim, Min Choi, Muntajin Rahman, Jinwon Choi, Kiryang Kim, Seungjoon Moon, Mohammed Dalli, Rony Abdi Syahputra, Sang-Won Shin, Abdel Halim Harrath, Moon Nyeo Park, Bonglee Kim, Hwa-Seung Yoo

**Affiliations:** ^1^ College of Korean Medicine, Kyung Hee University, Seoul, Republic of Korea; ^2^ East West Cancer Center, Seoul Korean Medicine Hospital, Daejeon University, Seoul, Republic of Korea; ^3^ Department of Oncology, Karmanos Cancer Institute, School of Medicine, Wayne State University, Detroit, MI, United States; ^4^ Chansol Hospital of Korean Medicine, Incheon, Republic of Korea; ^5^ Laboratory of Bioresources, Biotechnology, Ethnopharmacology and Health, Faculty of Sciences, University Mohammed the First, Oujda, Morocco; ^6^ Higher Institute of Nursing Professions and Health Techniques, Oujda, Morocco; ^7^ Department of Pharmacology, Faculty of Pharmacy, Universitas Sumatera Utara, Medan, Sumatera Utara, Indonesia; ^8^ Department of Humanities & Social Medicine, School of Korean Medicine, Pusan National University, Yangsan-si, Gyeongsangnam-do, Republic of Korea; ^9^ College of Science, Department of Zoology, King Saud University, Riyadh, Saudi Arabia

**Keywords:** *Atractylodes lancea* DC., prostate cancer, reactive oxygen species, apoptosis, mitochondrial membrane potential

## Abstract

**Introduction:**

Prostate cancer (PCa) is a malignancy characterized by abnormal cell proliferation in the prostate gland, a critical component of the male reproductive system. Atractylodes lancea DC. (ALD), a medicinal herb commonly used in traditional Asian medicine, is highly regarded for its antioxidant, antidiabetic, and anticancer properties. Virtual docking stud-ies have identified Atractylenolide II and III as active components of ALD, demonstrating strong binding potential to inhibit androgen receptor (AR) activity, with docking scores of -8.9 and -9.3, respectively. These findings suggest that ALD may exert a synergistic effect comparable to or greater than that of enzalutamide (ENZ) in inhibiting AR. How-ever, its specific anticancer and anti-metastatic mechanisms in prostate cancer remain unclear.

**Methods:**

The cytotoxic effects of ALD were evaluated on PC3 and DU145 prostate cancer cells, as well as on the normal prostate cell line BPH-1. Cell viability was assessed using the EZ-Cytotoxic kit, while colony formation assays and TUNEL staining were used to meas-ure proliferation and apoptosis, respectively. Apoptosis was further analyzed through an-nexin V-FITC/PI staining and quantified by flow cytometry (FACS). Western blotting was performed to elucidate the underlying molecular mechanisms. Additionally, mito-chondrial membrane potential (ΔΨm) and intracellular calcium levels were measured to evaluate mitochondrial function, while reactive oxygen species (ROS) generation was assessed with and without pretreatment with N-acetylcysteine (NAC) .

**Results:**

ALD selectively reduced the viability of PC3 and DU145 prostate cancer cells while spar-ing BPH-1 normal prostate cells, demonstrating cancer-selective cytotoxicity. ALD dis-rupted mitochondrial function by reducing ΔΨm and increasing intracellular calcium lev-els. A concentration-dependent increase in ROS generation was observed in PC3 and DU145 cells, which was completely inhibited by NAC pretreatment, confirming a ROS-mediated mechanism. Colony formation assays revealed a significant reduction in prolif-eration, while TUNEL and annexin V-FITC/PI staining indicated enhanced apoptosis. Western blot analysis showed that ALD modulates critical survival pathways, leading to apoptotic cell death.

**Discussion:**

These findings demonstrate that ALD exerts potent anticancer effects against metastatic prostate cancer cells through ROS-mediated apoptosis and mitochondrial dysfunction, while exhibiting minimal cytotoxicity toward normal prostate cells. The presence of ac-tive compounds such as Atractylenolide II and III suggests a synergistic interaction that enhances AR inhibition and promotes apoptosis. ALD’s ability to engage multiple path-ways highlights its therapeutic potential as a selective and multifaceted treatment for ag-gressive prostate cancer.

## 1 Introduction

Prostate cancer (PCa), a malignancy originating in the prostate gland, a small organ responsible for producing seminal fluid to support and transport sperm, is among the most prevalent cancers affecting men worldwide (1). Prostate cancer is more prevalent in industrialized nations, with GLOBOCAN estimating 1,276,106 new cases and 358,989 deaths worldwide in 2018. Each year, approximately 80,000 individuals globally succumb to prostate cancer, with an average of 190,000 new cases diagnosed annually (2). In Korea, recent data ranks prostate cancer as the third most commonly diagnosed cancer in men, highlighting a pressing need for effective prevention and treatment strategies (3, 4). The Korean Society of Medical Oncology (KSMO) established comprehensive guidelines in 2017 for managing metastatic prostate cancer, underscoring the increasing national and global concern regarding its impact (5). Understanding incidence trends and prognosis is essential, as outcomes are influenced by factors such as cancer stage, individual health, and response to therapy (6). While some prostate cancer cases progress slowly, allowing patients to live for many years with minimal health effects, the continuous advancement of therapies aims to improve survival and quality of life (7). Therefore, identifying novel therapies with minimal adverse effects is crucial for enhancing patient outcomes. Traditional Chinese Medicine (TCM) is an invaluable source for discovering new cancer treatments, with many herbs showing significant pharmacological properties (8). Among these, *Atractylodes lancea* DC. (ALD) stands out due to its wide-ranging effects, including antioxidant, antidiabetic, and anticancer activities (9). However, the precise mechanisms by which ALD exerts anticancer and anti-metastatic effects, particularly against PCa, remain underexplored. PCa progression is strongly linked to the androgen receptor (AR) signaling pathway, which promotes the growth and survival of cancerous cells (10). Advanced PCa treatment often involves androgen deprivation therapy (ADT) to reduce androgen levels or inhibit AR function (11). The AR plays a critical role in prostate gland development and in driving PCa progression by facilitating cell growth, survival, and migration (12). Virtual docking studies have revealed that components of TCM, including Atractylenolide II and III, possess the potential to bind and inhibit AR ligand activity, indicating a possible therapeutic pathway (13). Furthermore, *in vitro* and *in vivo* studies have demonstrated the therapeutic efficacy of ALD and its bioactive compounds, including atractylodin and β-eudesmol, in treating cholangiocarcinoma. To further assess the immunomodulatory effects of ALD in humans, a randomized, double-blind, placebo-controlled phase I clinical trial was conducted, providing additional insights into its clinical potential (14). Recent studies underscore Atractylenolide II as a powerful inducer of apoptosis in prostate cancer cells. By effectively modulating the androgen receptor (AR) and JAK2/STAT3 signaling pathways, it emerges as a compelling targeted therapeutic strategy, making it a promising candidate for treating metastatic prostate cancer (15). These insights underscore the need for further research on the therapeutic potential of ALD against PCa and inspired our investigation into its ability to trigger apoptosis in prostate cancer cells by disrupting AR signaling and other critical survival pathways. Mechanisms that selectively induce cancer cell death hold great promise for targeted therapies (16). Apoptosis, a programmed cell death process, is central to developing effective anticancer drugs (17, 18). Disruption of mitochondrial function vital for cellular energy production and apoptosis regulation represents a powerful approach to inducing cancer cell death (19). Research increasingly shows that mitochondrial ROS production is a key trigger of apoptosis (20). Elevated ROS levels disturb cellular homeostasis, activating signaling pathways that lead to cell death (21). Thus, agents that promote mitochondrial ROS generation are considered promising therapeutic candidates. In this study, we explore the hypothesis that ALD exerts anticancer effects by inducing ROS-mediated apoptosis in prostate cancer cells. Using PC3 and DU145 cell lines, established models for PCa research, we examined ALD’s effects on cell viability, mitochondrial activity, ROS production, and apoptotic protein expression. Through well-established *in vitro* assays, we evaluated ALD’s impact on cell survival, colony formation, and key apoptotic markers, as well as its influence on mitochondrial function and ROS generation. This research aims to advance the development of novel therapies for metastatic prostate cancer by elucidating the mechanisms underlying ALD’s anticancer effects.

## 2 Materials and methods

### 2.1 Preparation of *Atractylodes Lancea* DC. extract

In Gangwon Province, Korea, *Atractylodes lancea* DC. (ALD) was collected (200 g), and a voucher specimen (Registration number BK059) was preserved in the herbarium of the Cancer Molecular Targeted Herbal Research Center at Kyung Hee University. Following established protocols (22), dried ALD was extracted in 100% ethanol, concentrating the solution to 100 mL using an evaporator at 98°C for 24 hours. After lyophilization, the extraction yield was 18%, resulting in a powdered form. ALD was prepared as a stock solution at a concentration of 200 mg/mL in 100% dimethyl sulfoxide and stored at -20°C for future use.

### 2.2 Culturing of cell lines

PC3 (a prostate cancer cell line with high metastatic potential) and DU145 cells were obtained from the Korean Cell Line Bank (KCLB) and cultured in RPMI 1640 medium supplemented with 10% fetal bovine serum (FBS), 2 mM L-glutamine, and 10,000 units/mL of penicillin and streptomycin (Gibco, Grand Island, NY, USA). The culture medium was refreshed every two to three days. The BPH-1 cell line (ATCC, PCS-440-030; Human Benign Prostatic Hyperplasia epithelial cells) was also cultured in RPMI 1640 medium with 10% FBS, 2 mM L-glutamine, and 10,000 units/mL of penicillin and streptomycin.

### 2.3 Cytotoxicity evaluation assay

Cell viability was assessed using a cytotoxicity assay. PC3, DU145 and BPH-1 cells were plated at a density of 1×10⁴ cells per well in 96-well plates and treated with ALD at varying concentrations (12.5, 25, 50, 100, or 200 μg/mL) for 24, and 48 hours. At each time point, cell viability was evaluated using a cell viability assay kit according to the manufacturer’s instructions (Daeil Lab Service, Seoul, Korea). Absorbance was measured at 450 nm using a microplate reader (Bio-Rad, Hercules, CA, USA) to determine cellular viability.

### 2.4 Colony formation assay

The colony formation assay was conducted to evaluate cell proliferation and the effects of ALD treatment on prostate cancer cells. PC3 and DU145 cells were seeded in triplicate into 6-well plates at a density of 1000 cells per well and treated with 50 and 100 μg/mL of ALD for 24 hours. After treatment, the cells were cultured in fresh growth medium for 9 days, with the medium replaced every two days, and maintained at 37°C in a 5% CO₂ incubator. Colony formation was assessed by staining: cells were fixed in 70% ethanol for 10 minutes and stained with 0.5% crystal violet to visualize colonies. Colonies consisting of at least 50 cells were counted as individual colonies using ImageJ software (National Institutes of Health, Bethesda, Maryland, USA).

### 2.5 Reactive oxygen species detection assay

To assess ROS production, PC3 and DU145 cells were first incubated with 20 µM 2’,7’-Dichlorofluorescein diacetate (DCFDA) for 45 minutes. Following this, cells were treated with ALD at concentrations of 50 and 100 µg/mL for 4 hours. Fluorescence was measured in a 96-well plate format using an ELISA reader (Bio-Rad, Hercules, CA, USA) at excitation/emission wavelengths of 490/595 nm to confirm ROS generation. For further validation, cells were treated with ALD for 24 hours and then incubated with 5 mM N-acetylcysteine (NAC) as a ROS inhibitor, followed by 30 minutes of DCFDA staining (20 µM). Fluorescence was then analyzed using a flow cytometry analysis (FACS) Calibur flow cytometer (Becton Dickinson, Bergen County, NJ, USA) to measure ROS levels and evaluate NAC’s inhibitory effects on ALD-induced ROS production. This dual approach, using both ELISA and FACS, provided comprehensive confirmation of ROS activity in response to ALD treatment.

### 2.6 Analysis of mitochondrial membrane potential

PC3 and DU145 cells were incubated with JC-1 dye (20 µM) for 45 minutes to assess mitochondrial membrane potential (MMP), followed by treatment with ALD at concentrations of 50 and 100 µg/mL for 4 hours. The JC-1 dye, which differentiates between healthy and depolarized mitochondria based on fluorescence, was used to monitor mitochondrial health (Mitochondria Function Assay Kit, Thermo-Fisher Scientific, USA). MMP was evaluated by measuring fluorescence from JC-1 aggregates (intact mitochondria, *λ*_ex_/*λ*_em_ = 535/595 nm) and JC-1 monomers (depolarized mitochondria, *λ*_ex_/*λ*_em_ = 475/535 nm). This ratio provides insights into ALD’s impact on mitochondrial function in PC3 and DU145 cells. For validation, we included a positive control using 2 µM FCCP (carbonyl cyanide 4-(trifluoromethoxy)phenylhydrazone), a known mitochondrial uncoupler, and analyzed the results with a FACS Calibur flow cytometer (Becton Dickinson, Bergen County, NJ, USA). Consistent gating for dye exclusion was applied across experiments to accurately reflect changes in mitochondrial health under different treatment conditions.

### 2.7 Cytosolic Ca^2+^ quantification assay

Cells were treated with ALD for 24 hours. After treatment, a chromogenic reagent and calcium buffer were added to each well and incubated for an additional 10 minutes. Absorbance was then measured at 575 nm using a microplate reader (Bio-Rad, Hercules, CA, USA) to quantify the response.

### 2.8 TUNEL apoptosis detection assay

PC3 and DU145 cells were seeded onto a 4-well culture slide (SPL, Pocheon, Republic of Korea) and treated with ALD for 24 hours. After treatment, cells were washed with phosphate-buffered saline (PBS), fixed with 4% paraformaldehyde (Bylabs, San Francisco, CA, USA), and permeabilized using 0.2% Triton X-100 solution (Promega, Madison, WI, USA), followed by additional PBS washes. Cell apoptosis was then analyzed using the DeadEnd™ Fluorometric TUNEL System (Promega, Madison, WI, USA). After treatment with equilibrium buffer for 10 minutes, the Nucleotide Mix and rTdT enzyme were added, and samples were incubated for one hour. Following a 15-minute reaction, samples were washed with 2× SSC solution and PBS, stained with 1 μg/mL DAPI, mounted, and visualized using a Zeiss LSM 800 confocal microscope (Zeiss, Oberkochen, Germany). To further assess apoptosis, annexin V-FITC labeling was employed. PC3 and DU145 cells were plated in a six-well plate and incubated for 24 hours at 37°C. Cells were then treated with ALD for 24 hours, harvested, and incubated with 5 µL annexin V-FITC on ice at 4°C for 15 minutes. After labeling, cells were centrifuged at 1000 g for 5 minutes. Apoptosis was quantified using a FACS Calibur flow cytometer (Becton Dickinson, Bergen County, NJ, USA), providing an accurate measurement of apoptotic cell populations.

### 2.9 Protein expression analysis by western blot analysis

After treating PC3 and DU145 cells with ALD at concentrations of 50 and 100 µg/mL for 24 hours, the cells were lysed on ice for 30 minutes using a lysis buffer containing protease inhibitors (Translab, Daejeon, Korea). The cell lysates were then centrifuged at 13,000 rpm for 10 minutes to collect the supernatant containing the proteins. Protein concentrations were determined using the BSA assay to ensure equal protein loading. Equal amounts of protein were loaded onto 6–15% SDS-PAGE gels and separated by electrophoresis at 100 V for 100 minutes. Proteins were then transferred onto nitrocellulose membranes at 300 mA for 120 minutes. Following the transfer, membranes were rinsed three times for 10 minutes each with TBS containing 0.1% Tween 20 (TBST) and then blocked with 5% skim milk dissolved in TBST for 1 hour at room temperature. The membranes were incubated overnight at 4°C with specific primary antibodies diluted 1:10,000, including pro-PARP, cleaved PARP (c-PARP), Bcl-2, survivin, cytochrome c, ATF4, TGF-β, DNMT1, N-Cadherin, and E-Cadherin (Cell Signaling Technology, Beverly, MA, USA). After washing with TBST, membranes were incubated with appropriate HRP-conjugated secondary antibodies either anti-mouse (Abcam, Cambridge, UK) or anti-rabbit (Bioss Antibodies, Woburn, MA, USA) for 1 hour at room temperature. Membranes were washed again with TBST, and protein bands were visualized using chemiluminescence detection reagents (Davinch-K, Seoul, Korea) and scanned for analysis.

### 2.10 Wound healing assay

The cell lines PC3 and DU145 were used for the wound healing assay. Cells were plated into 6-well plates at a density of 1 × 10⁶ cells per well and cultured in RPMI complete medium for 24 hours to reach approximately 90% confluence. A scratch was made across the cell monolayer using a 200 µL pipette tip to create a wound gap. Detached cells were removed by gently washing with PBS. The cells were then treated with ALD for a 24 hour intervention period. Following this treatment, the ALD-containing medium was replaced with fresh complete medium, and images of the wound closure were taken to assess the effects of ALD on cell migration.

### 2.11 Statistical analysis

Statistical analyses were conducted using one-way ANOVA followed by Tukey’s *post-hoc* test for multiple group comparisons to determine statistically significant differences between experimental groups. Most experiments were performed in triplicate (n=3), and results are expressed as mean ± standard deviation (SD). For direct comparisons between control and treated groups, Student’s t-test was applied where appropriate. Statistical analyses were conducted using SigmaPlot version 12 software (SysTest Software Inc., San Jose, CA, USA), and p-values were considered statically significant as follows p* < 0.05, p** < 0.01, *p* ***< 0.001 were considered statically significant.

## 3 Results

### 3.1 The active components of ALD and their targeted pathways in prostate cancer

**Figure 1:**
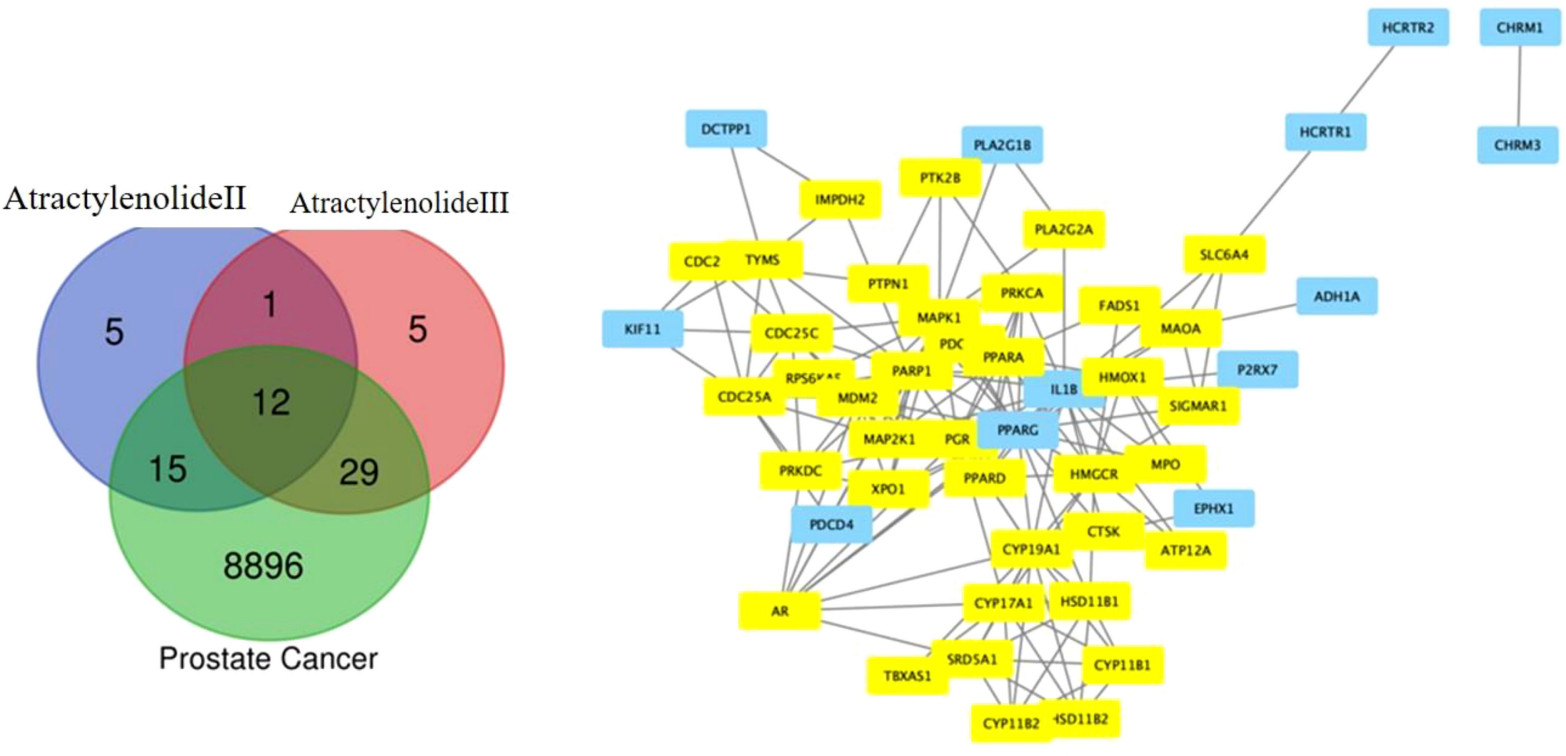
Venn Diagram of Target Overlaps. The Venn diagram illustrates the overlapping targets between Atractylenolide II, Atractylenolide III, and known prostate cancer markers, highlighting shared molecular targets. This overlap suggests potential pathways through which these compounds may exert anticancer effects in prostate cancer.

Active components and potential targets 8896 and 56 for Atractylenolide II and Atractylenolide III, respectively, were identified using the TCMSP, TCMIP, and BATMAN-TCM databases (23). To further understand the molecular interactions, KEGG pathway enrichment analysis and Gene Ontology (GO) annotation were performed, helping to reveal the core features and functions of central genes within these networks (24). In this network, the AR emerged as a central node, underscoring its critical role in regulating key pathways involved in prostate cancer progression. The central positioning of AR within this pathway network highlights its pivotal role in prostate cancer biology, particularly in androgen-dependent signaling. The AR, known as a primary mediator in prostate cancer and male reproductive tissue maintenance, functions by binding to androgens, male sex hormones, and subsequently acting as a transcription factor to regulate gene expression. Surrounding AR, the network consists of genes and proteins that either directly interact with it or are components of pathways affected by AR activity. For instance, genes such as *CYP19A1* and *HSD17B1*, involved in steroid metabolism, can influence androgen levels, thus modulating AR activation. Other components, like MAPK1 and SRC, participate in signaling cascades that can enhance or modify AR activity, impacting cellular responses to its activation. As illustrated in **Figure 1**, the interconnected network surrounding AR reinforces its critical role in the regulatory pathways involved in prostate cancer, demonstrating how key signaling proteins and metabolic enzymes converge to influence AR-mediated transcription and, ultimately, prostate cancer cell proliferation and survival.

### 3.2 Molecular docking analysis of ligands interactions with the androgen receptor

**Figure 2:**
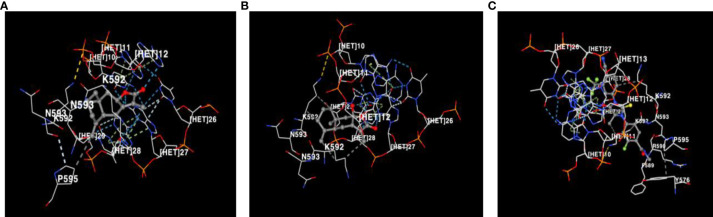
Visualization of amino acid residue interactions with **(A)** Atractylenolide II, **(B)** Atractylenolide III, and **(C)** Enzalutamide (ENZ). Interactions between amino acid residues of the androgen receptor and the ligands Atractylenolide II, Atractylenolide III, and ENZ are shown, highlighting the binding affinity and key contact points for each compound.

Molecular docking analysis revealed significant interactions between the androgen receptor (PDB ID: 1r4i), a key target in prostate cancer, and three ligands: Atractylenolide II, Atractylenolide III, and Enzalutamide (ENZ). Docking scores, which indicate binding affinity, were -8.9, -9.3, and -10.0, respectively, as shown in **Table 1**. These scores suggest that all three compounds exhibit strong binding tendencies, with ENZ demonstrating the highest affinity. Notably, we highlight that the combined docking scores for Atractylenolide II and III suggest a potential interaction strength that may match or even surpass that of ENZ. This supports the concept that multiple bioactive constituents in herbal formulations like ALD can work synergistically to enhance biological effects, resulting in broader and more stable receptor binding. As illustrated in **Figure 2**, each ligand formed detailed interaction profiles with specific amino acid residues on the androgen receptor. Atractylenolide II engaged with multiple residues on Chain A, including LYS563, VAL564, LYS567, and ARG590, and with ARG568, GLN574, and TYR576 on Chain B, among others. Atractylenolide III showed a similar pattern, interacting with residues like TYR554, LYS563, and ASN593 on Chain A and with LYS588 and LYS592 on Chain B. ENZ, showing the highest binding affinity, formed interactions with critical residues such as VAL564, LYS567, and PHE589 in Chain A, as well as additional binding with ARG568, TYR576, and LEU577 in Chain B. These findings indicate strong and specific interactions between these ligands and the androgen receptor, with ENZ showing the most robust profile. However, the cumulative binding potential of Atractylenolide II and III may offer a multi-faceted interaction strategy, reinforcing the therapeutic value of ALD in targeting prostate cancer through enhanced receptor engagement.

**Table 1:** Ligand interactions with the androgen receptor as a prostate cancer target marker (PDB ID: 1r4i).

Ligands	Docking Score	Amino Acid recidue interaction
atractylenolideII	-8.9	Chain A: LYS563 VAL564 LYS567 ARG568 LYS588 PHE589 ARG590 ARG591 LYS592 ASN593 PRO595 Chain B: ARG568 GLN574 LYS575 TYR576 LEU577 LYS592 ASN593 PRO595
atractylenolideIII	-9.3	Chain A: TYR554 LYS563 VAL564 LYS567 ARG568 LYS588 PHE589 ARG591 LYS592 ASN593 Chain B: LYS588 LYS592 ASN593
Enzalutamide	-10.0	Chain A: VAL564 LYS567 ARG568 LYS588 PHE589 ARG590 ARG591 LYS592 ASN593 CYS594 PRO595 Chain B: PHE565 ARG568 ALA569 GLN574 LYS575 TYR576 LEU577 LYS592 ASN593 CYS594 PRO595

### 3.3 ALD demonstrated potent cytotoxic activity against prostate cancer cells

**Figure 3:**
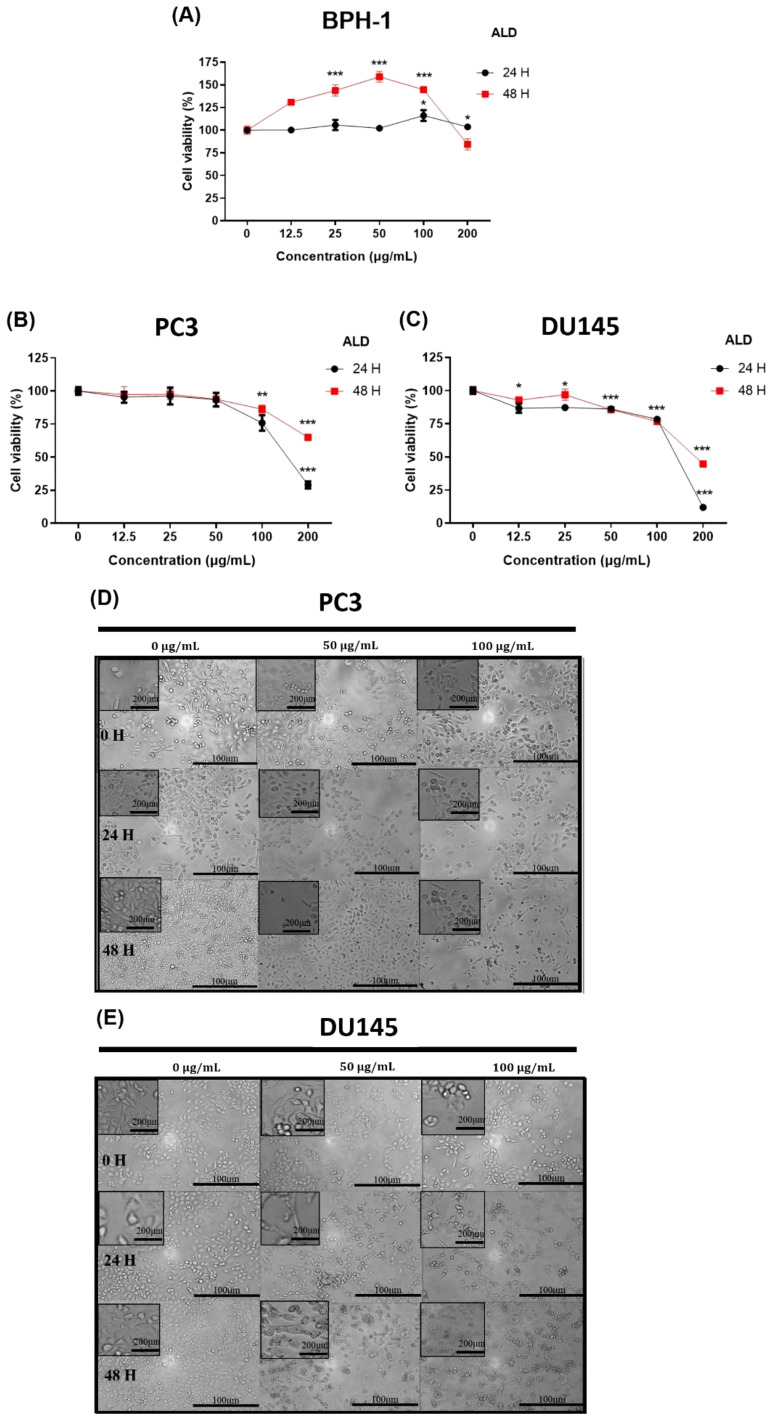
Cytotoxic effects of ALD on PC3 and DU145 cell lines. **(A–C)** BPH-1, PC3, and DU145 cells were treated with ALD at various concentrations (12.5, 25, 50, 100, and 200 μg/mL) for 24, and 48 hours in 96-well plates. Cell viability was assessed using EZ-Cytox, a cell viability assay reagent. Data represent the means of three independent experiments and are shown as Means ± SD; p* < 0.05, p** < 0.01, and p*** < 0.001 compared to control. **(D, E)** Effect of ALD on the morphology of prostate cancer cells. PC3 and DU145 cells (250,000 cells/well) were seeded into 6-well plates and treated with ALD at concentrations of 50 and 100 μg/mL for 24 and 48 hours. Images were captured using phase-contrast microscopy at 100× magnification. Scale bars = 100 and 200 μm.

The cytotoxic effects in prostate cancer cells refer to the ability of a compound or treatment to induce cell death or suppress the proliferation of these malignant cells. Understanding and refining these cytotoxic mechanisms is a critical focus in advancing potent cancer therapies, as it holds the potential to selectively target and eradicate cancer cells while preserving healthy tissue (25). The cytotoxic effects of ALD on prostate cancer and normal cells were assessed at concentrations of 12.5, 25, 50, 100, and 200 μg/mL across 24 and 48 hours. **Figures 3A–C** demonstrate that ALD effectively inhibits the proliferation of PC3 and DU145 prostate cancer cells in a dose- and time-dependent manner, while exhibiting minimal impact on BPH1 normal prostate cells. This selective inhibition underscores ALD’s potential as a targeted therapeutic agent with specificity toward cancerous cells, sparing healthy prostate cells. These findings, illustrated in **Figures 3A–C** underscore ALD’s potential as a targeted therapeutic agent for prostate cancer. To further investigate its cytotoxic impact, cells were treated with ALD at 50 and 100 μg/mL, revealing pronounced cell death characteristics as early as 24 hours treatment. **Figures 3D, E** vividly capture these morphological changes, underscoring ALD’s potent and rapid cytotoxic effects on prostate cancer cells. Distinctive apoptotic features, including plasma membrane disintegration and the formation of apoptotic bodies, were prominently observed in ALD-treated cells. In contrast, untreated PC3 and DU145 cells remained viable up to 48 hours, displaying increased cell size, cell number, and a progressively flatter morphology over time. ALD-treated PC3 and DU145 cells, however, exhibited consistent and striking apoptotic characteristics, with cell death morphology becoming more pronounced in a dose-dependent manner.

### 3.4 ALD suppressed colony formation in prostate cancer cells

**Figure 4:**
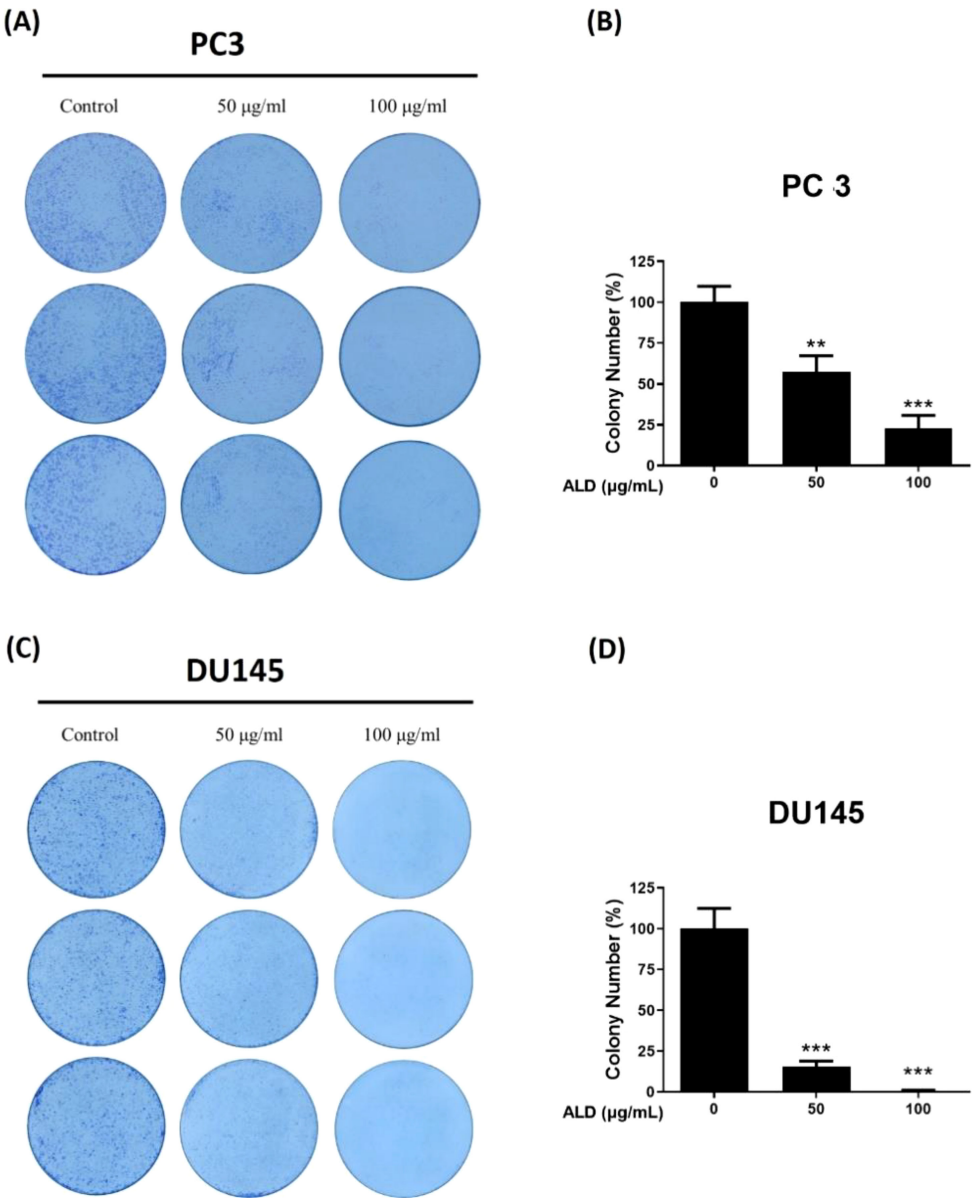
ALD inhibits cell proliferation in PC3 and DU145 cells. **(A, B)** PC3 and **(C, D)** DU145 cells were seeded at a density of 1000 cells/well in 6-well plates and allowed to adhere for 24 hours. Cells were then treated with 50 and 100 µg/mL of ALD and cultured in fresh medium for 9 days, followed by fixation and staining with crystal violet to visualize colonies. Representative images of colony formation in PC3 cells (left) and quantitative analysis shown as a bar graph (right). Representative images of colony formation in DU145 cells (left) with corresponding quantitative analysis (right). Data are expressed as means ± SD; p** < 0.01, and p*** < 0.001 compared to control.

The clonogenic, or colony-forming cell (CFC) assay, is widely regarded as the gold-standard quantitative method for assessing the proliferative capacity of progenitor cells *in vitro* by measuring the ability of a single cell to survive, proliferate, and form a colony (26). To investigate the antiproliferative effects of ALD on prostate cancer cells, a colony formation assay was performed with PC3 and DU145 cell lines cultured on 6-well plates, both in the presence and absence of ALD, over a 9-day period. ALD treatment at a concentration of 100 µg/ml significantly inhibited colony formation in both cell lines (**Figure 4**), underscoring its strong suppressive effect on the proliferation of PC3 and DU145 cells. These results highlight ALD’s potent antiproliferative action against prostate cancer cells, further validating its potential as an effective therapeutic agent.

### 3.5 ALD triggered apoptosis through Ca^2+^ uptake and mitochondrial membrane potential disruption in prostate cancer cells

**Figure 5:**
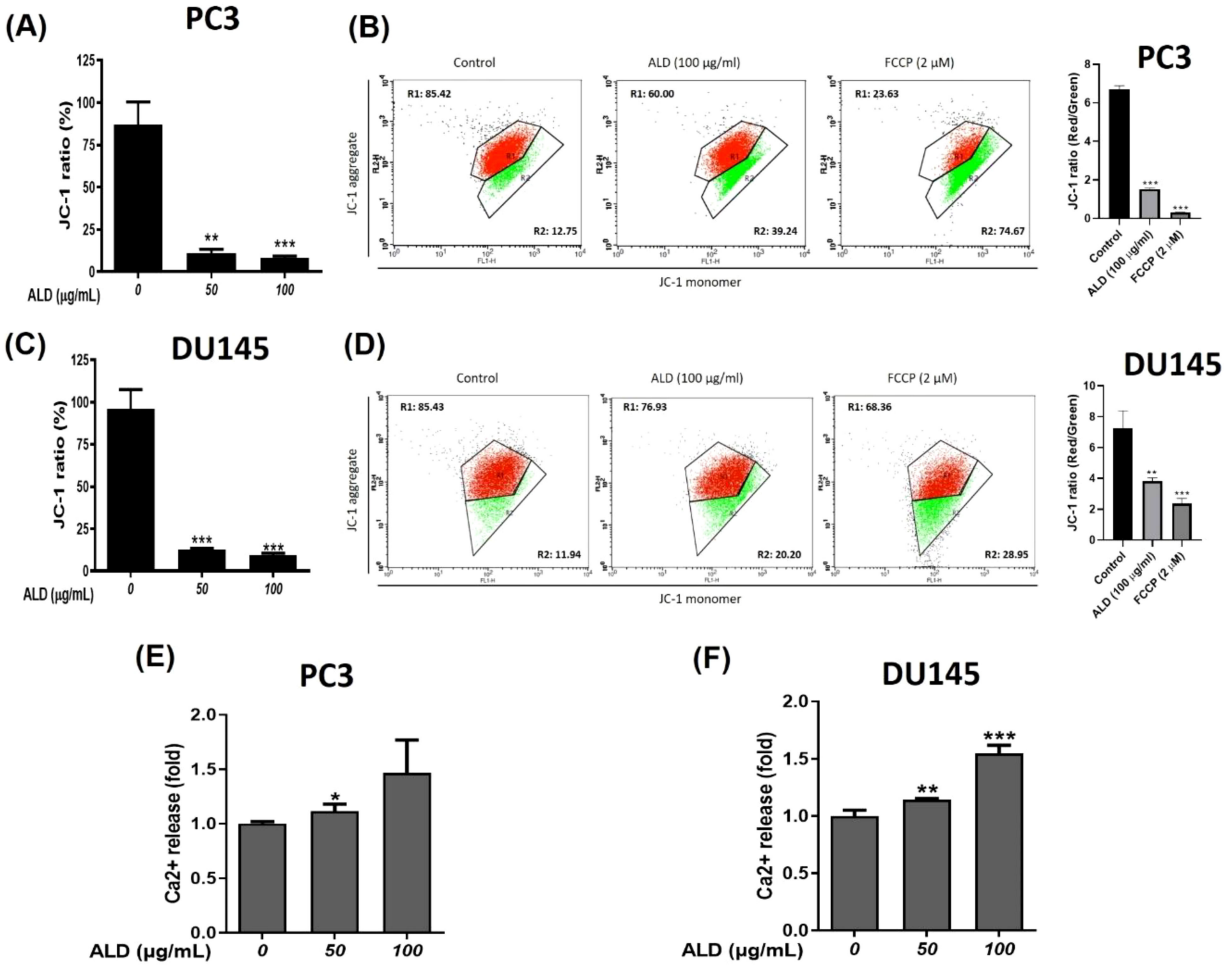
ALD-Induced Mitochondrial Dysfunction in PC3 and DU145 Cells. **(A–C)** Mitochondrial membrane potential in PC3 and DU145 cells was assessed after 4 hours of treatment with ALD at concentrations of 50 and 100 µg/ml. Cells were stained with JC-1 dye, and the fluorescence ratio of JC-1 aggregates (indicative of healthy mitochondria) to monomers (indicative of depolarized mitochondria) was analyzed. Data are presented as mean ± SD, with p** < 0.01 and p*** < 0.001 compared to the untreated group. **(B–D)** PC3 and DU145 cells were treated with ALD at a concentration of 100 µg/ml for 24 hours, with FCCP (2 µM) used as a positive control for mitochondrial depolarization. Mitochondrial membrane potential was assessed using JC-1 staining followed by FACS. The red-to-green fluorescence ratio was measured to evaluate changes in membrane potential, with a decrease indicating mitochondrial depolarization. Results are shown as mean ± SD, with p** < 0.01, and p*** < 0.001 versus the untreated group. **(E, F)** Cytosolic Ca²⁺ concentration in PC3 and DU145 cells was evaluated following 24 hours of ALD exposure at the same concentrations. Chemogenic reagent labeling was used, and fluorescence was measured with an ELISA reader. Results are shown as mean ± SD, with p* < 0.05, p** < 0.01, and p*** < 0.001 versus the untreated group.

Disruption of the mitochondrial membrane triggers a cascade of apoptotic events, including a reduction in mitochondrial membrane potential (MMP) and an increase in cytoplasmic Ca^2+^ concentration (27). Effective anticancer agents are known to induce apoptosis in cancer cells by elevating intracellular Ca^2+^ levels, a hallmark of mitochondrial dysfunction. In our study, we used the JC-1 assay to assess changes in MMP, which revealed a notable decrease in MMP (28) in ALD-treated PC3 and DU145 cells at 50 and 100 µg/ml concentrations. This was indicated by a reduced red-to-green fluorescence ratio in apoptotic cells (**Figures 5A, C**). Treatment with FCCP, a known disruptor of mitochondrial function, confirmed that the observed reduction in oxygen consumption suggests irreversible mitochondrial impairment within certain treatment ranges (29). To further validate the JC-1 assay results, flow cytometry (FACS) analysis was conducted using FCCP as a positive control. The FACS results supported the JC-1 findings, showing that ALD treatment significantly reduced the red-to-green fluorescence ratio, indicating disrupted mitochondrial membrane potential. Interestingly, this reduction in the ALD-treated group was comparable to the effect observed with FCCP, the positive control, suggesting that ALD’s effect on mitochondrial function is similar to that of FCCP in both PC3 and DU145 cells. This reinforces the potent effect of ALD in altering mitochondrial dynamics, as illustrated in **Figures 5B, D**. Given that changes in MMP can impact intracellular ion homeostasis, we also measured Ca^2+^ levels in ALD-treated PC3 and DU145 cells, which revealed a concentration-dependent increase in Ca^2+^ levels in both cell lines (**Figures 5C, D**). This rise in Ca^2+^ supports the hypothesis that ALD induces apoptosis through mitochondrial destabilization. These findings align with previous studies suggesting that decreased MMP and elevated intracellular Ca^2+^ are essential mechanisms in apoptosis induced by anticancer agents. Future research should further investigate the specific molecular pathways through which ALD exerts these effects and explore its therapeutic potential in cancer treatment.

### 3.6 ALD enhanced reactive oxygen species production and induced apoptosis in prostate cancer cells

**Figure 6:**
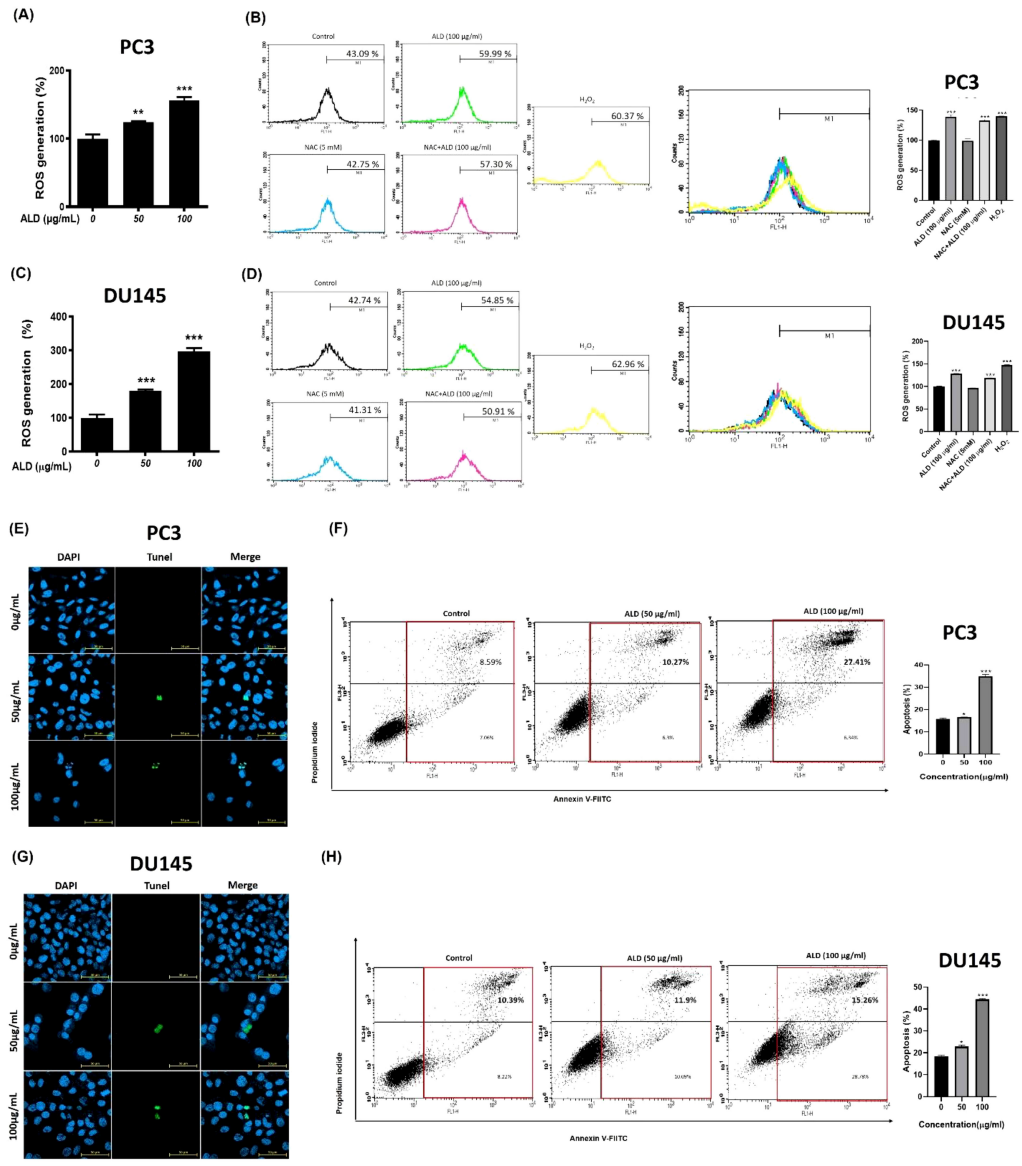
ALD increased ROS-mediated apoptosis in prostate cancer cells. **(A–C)** The cells were treated with ALD (50 and 100 µg/ml) for 4 hours in PC3 and DU145 cells. The reactive oxygen species generation results were analyzed using an oxidation-sensitive fluorescent dye. A 96-well microplate reader was employed to measure the fluorescence. Values represent the means of three experiments. Means ± SD; p*<0.05, p**<0.01 and p***<0.001 between two groups. **(B–D)** Flow cytometry analysis was conducted to measure ROS levels in PC3 and DU145cells treated with H_2_O_2_ and NAC. Cells were exposed to H_2_O_2_ to induce oxidative stress, while NAC was used to evaluate its protective effects. ROS levels were quantified and compared between treated and control groups. (Right) The quantitative analysis of FACS results is illustrated in the bar graph. Data are presented as mean ± SD, with a significant difference indicated by p***<0.001 when compared to the untreated control group. The presence of cell death. **(E–G)** PC3 and DU145 cells was confirmed using a confocal microscope. The nucleus emits blue fluorescence with a wavelength of 465 nm, while dead cells display fluorescent green images with a wavelength of 520 nm. Scar bar = 50μm. **(F–H)** Apoptotic rate of prostate cancer cells was assessed using Annexin V-FITC staining after a 24-hour treatment with 100 µg/mL of ALD. The results indicate the proportion of cells undergoing apoptosis in response to the treatment. (Right) The quantitative analysis of FACS results is illustrated in the bar graph. Data are presented as mean ± SD, with a significant difference indicated by p*<0.05, and p***<0.001 when compared to the untreated control group.

Reactive oxygen species (ROS) are known to drive cancer progression by damaging DNA through oxidation, promoting lipid peroxidation, and causing oxidative modifications in proteins, which can lead to structural DNA changes (30). To investigate whether ROS generation and DNA damage are central to ALD’s anticancer effects, we conducted DCFDA analysis and TUNEL assays on PC3 and DU145 cells treated with ALD at 50 and 100 µg/mL. ALD treatment led to a significant, dose-dependent increase in ROS production in both cell lines, suggesting that ROS is a critical mediator of ALD-induced apoptosis (**Figures 6A, C**). Higher ALD doses correlated with greater ROS generation, highlighting the role of oxidative stress (31) in ALD’s cytotoxic mechanism against prostate cancer cells. To further explore the role of specific ROS, such as superoxide anion (O_2_^−^), hydrogen peroxide (H_2_O_2_), and hydroxyl radicals (HO•), we used flow cytometry (FACS) to assess the impact of N-acetylcysteine (NAC), a ROS inhibitor, on ROS levels. Cells were treated with H_2_O_2_ to induce oxidative stress, with a subset also receiving NAC. FACS analysis, which used fluorescent probes to quantify ROS levels based on fluorescence intensity, showed that H2O2 treatment significantly elevated ROS levels compared to controls. Notably, ALD treatment induced ROS generation to a similar extent as H_2_O_2,_ suggesting that ROS plays a critical role in ALD’s apoptotic effects. Simultaneous NAC treatment mitigated the ROS increase, demonstrating NAC’s protective effect against oxidative stress and reinforcing ROS as a key mediator in ALD-induced apoptosis (**Figures 6B, D**). The TUNEL assay further supported these findings by revealing DNA fragmentation, a hallmark of apoptosis, in cells treated with ALD. TUNEL staining showed blue fluorescence marking nuclei and green fluorescence indicating fragmented DNA (**Figures 6E, G**). There was a positive correlation between ALD concentration and DNA fragmentation, confirming ALD’s role in inducing apoptosis through ROS-mediated DNA damage. Additionally, Annexin V/FITC assays demonstrated that ALD-induced apoptosis involves DNA damage, providing further evidence that ALD effectively triggers apoptosis in prostate cancer cells (**Figures 6F, H**).

### 3.7 ALD promoted mitochondrial-driven apoptosis in prostate cancer cells

**Figure 7:**
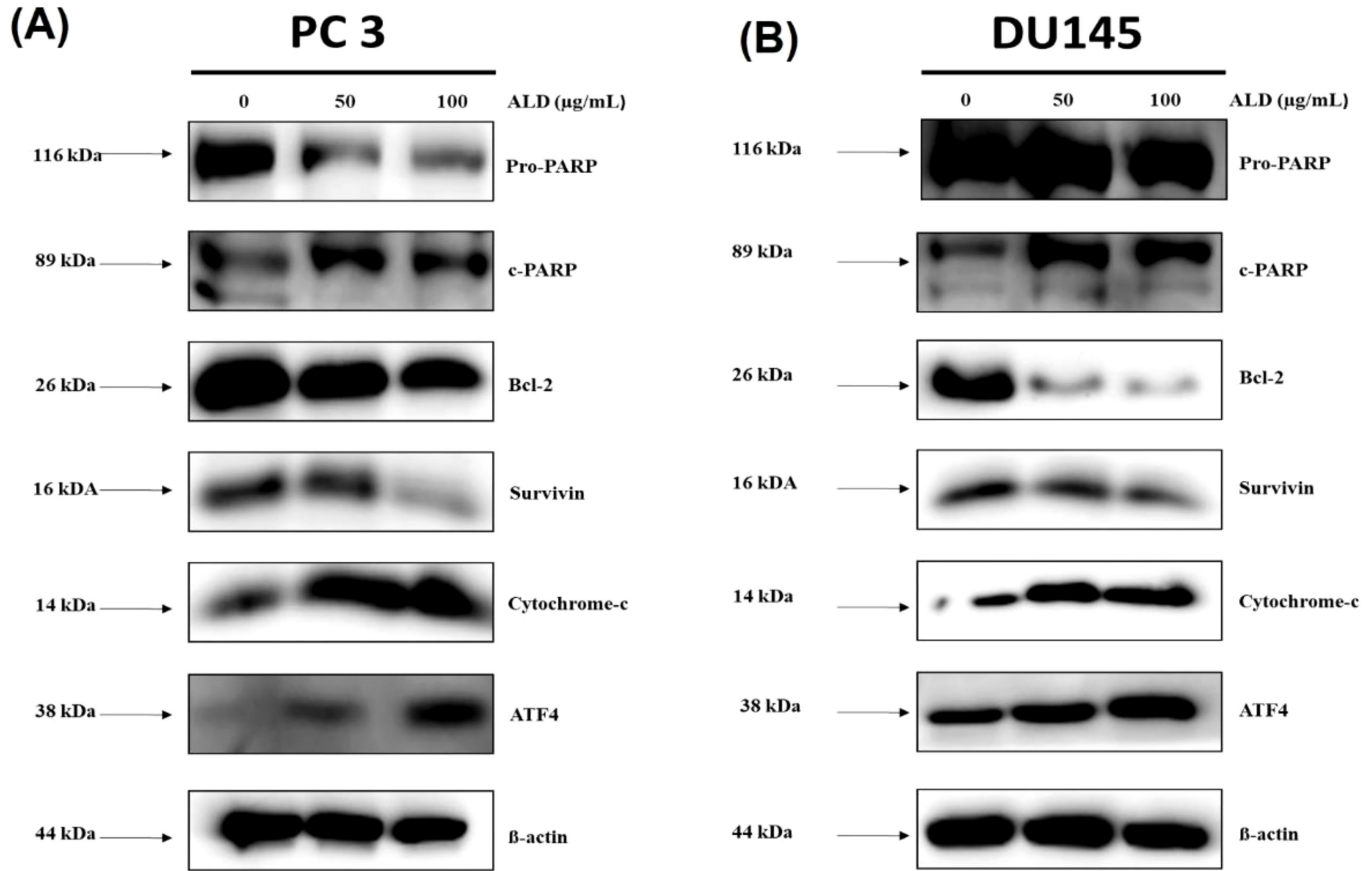
ALD enhances apoptosis pathway activation in PC3 and DU145 cells. **(A, B)** After a 24-hour treatment with specified doses of ALD, apoptosis-related protein expression was analyzed by western blotting in PC3 and DU145 cells. Probed proteins included pro-PARP, cleaved PARP (c-PARP), Bcl-2, survivin, cytochrome c, ATF4, and β-actin as a loading control, demonstrating ALD’s effect on apoptotic signaling pathways.

Cytochrome c, a mitochondrial protein, plays a crucial role in initiating apoptosis by activating caspases once released into the cytosol, thereby triggering a cascade that promotes programmed cell death (32). Elevated cytosolic cytochrome c levels indicate mitochondrial outer membrane permeabilization (MOMP), a pivotal early event in the apoptotic pathway (33). In addition, ATF4, a transcription factor responsive to cellular stress (34), especially the unfolded protein response (UPR) in the endoplasmic reticulum—signals increased stress levels and regulates genes associated with apoptosis (35). The cleavage of pro-PARP into c-PARP is another hallmark of apoptosis, as reduced levels of intact PARP confirm active apoptotic processes (36). Anti-apoptotic proteins like Bcl-2 prevent cytochrome c release by stabilizing the mitochondrial membrane (37), while survivin, an inhibitor of apoptosis protein (IAP), further supports cell survival by counteracting apoptotic signals (38). To confirm ALD’s role in promoting apoptosis and inhibiting cell growth, we treated PC3 and DU145 prostate cancer cells with increasing concentrations of ALD. Results demonstrated that ALD (50 and 100 µg/mL) significantly increased the levels of c-PARP, cytochrome c, and ATF4, while concurrently reducing the expression of pro-PARP, Bcl-2, and survivin in a dose-dependent manner, as shown in **Figures 7A, B**. This dose-dependent modulation of apoptosis-related proteins underscores ALD’s mechanism of action, enhancing pro-apoptotic signals while suppressing anti-apoptotic factors. Together, these findings suggest that ALD effectively induces apoptosis in prostate cancer cells by shifting the cellular balance toward cell death through an increase in pro-apoptotic proteins and a decrease in survival-promoting factors.

### 3.8 ALD suppressed EMT via modulation of TGF- β signaling in prostate cancer cells

**Figure 8:**
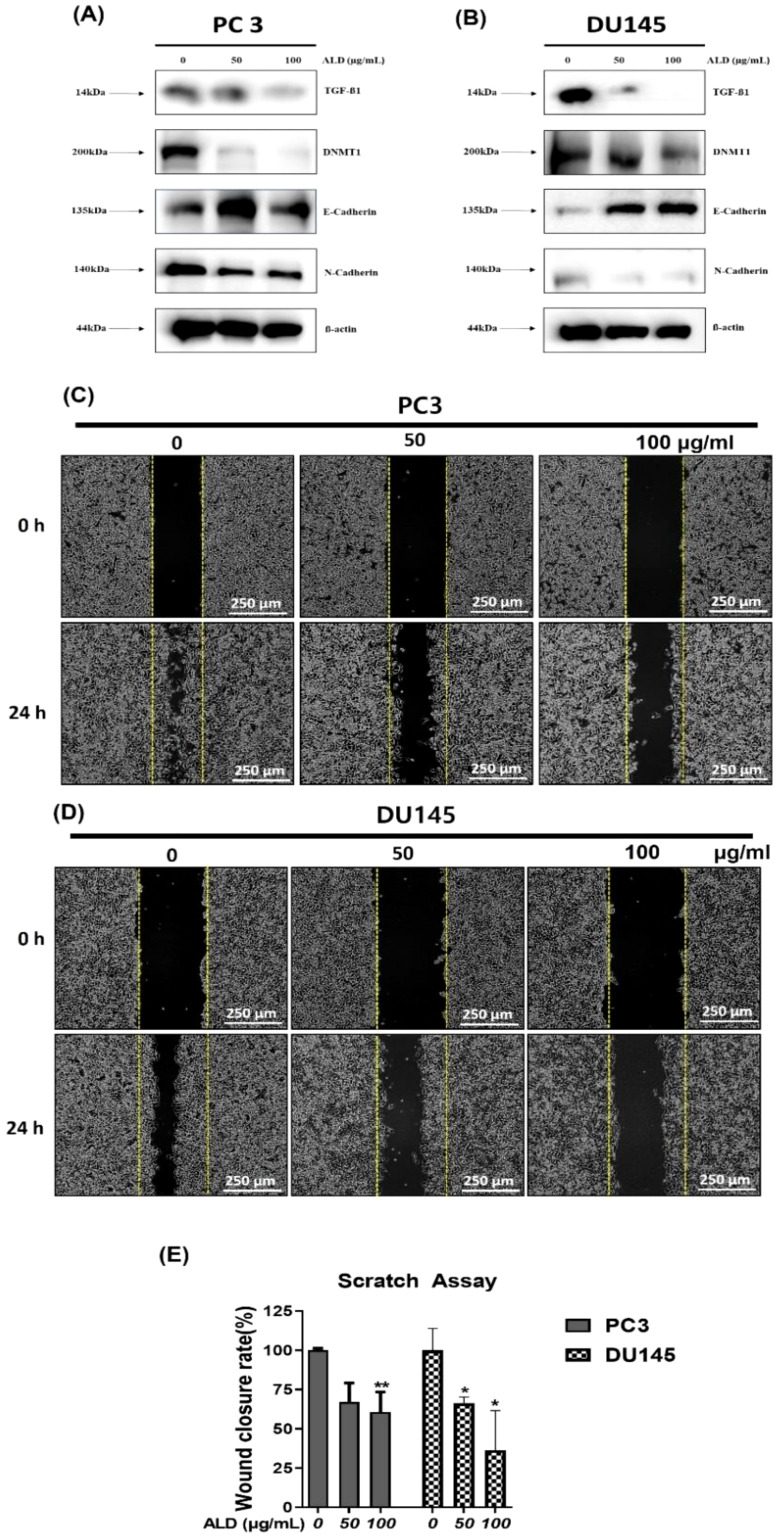
ALD inhibits EMT and cell migration in prostate cancer cells. **(A, B)** PC3 and DU145 cells were treated with ALD (50 and 100 µg/mL) or left untreated for 24 hours. Western blot analysis was performed to assess the expression of EMT-related proteins, including TGF-β, DNMT-1, E-cadherin, N-cadherin, with β-actin as the loading control. **(C, D)** Migration assay in PC3 and DU145 cells was conducted in 6-well plates to evaluate ALD’s effects on cell migratory behavior. **(E)** Quantitative analysis of cell migration is presented in the bar graph. Data are expressed as mean ± SD; p*<0.05, and p**<0.01 compared to the untreated control.

Androgens play a crucial role in promoting prostate cancer growth, with transforming growth factor-beta (TGF-β) known to synergize with androgens to facilitate the epithelial-to-mesenchymal transition (EMT) in prostate cancer cells (39). Furthermore, recent studies have highlighted DNMT1 as a key enhancer of tumor progression by promoting EMT (40). This transition is marked by a shift in cadherin expression: a decrease in E-cadherin (associated with cell adhesion) and an increase in N-cadherin (associated with motility), which collectively drive tumor invasion and metastasis (41). In this study, we investigated the effects of ALD on EMT-related proteins and cell migration in prostate cancer cells using western blot analysis (**Figures 8A, B**). ALD treatment led to a significant downregulation of TGF-β, N-cadherin, and DNMT1 levels, while upregulating E-cadherin expression in both PC3 and DU145 cells, suggesting a reversal of the EMT phenotype. Additionally, cell migration assays demonstrated that ALD treatment at concentrations of 50 μg/mL and 100 μg/mL significantly inhibited migration in PC3 and DU145 cells, respectively (**Figures 8C, D**). Western blotting results further confirmed this effect, showing a marked increase in E-cadherin alongside significant reductions in N-cadherin, Snail, and TGF-β levels in both cell lines (**Figures 8A, B**). These findings indicate that ALD effectively inhibits the EMT process, potentially reducing the metastatic capacity of prostate cancer cells. By modulating key EMT markers and impairing cell migration, ALD demonstrates significant potential as a therapeutic agent against metastatic prostate cancer.

## 4 Discussion

This study provides robust evidence that ALD, a widely used herb in traditional Chinese medicine, has significant therapeutic potential for prostate cancer. Through pharmacoinformatics, we examined ALD’s anticancer effects in PC3 and DU145 cells and found that ALD disrupts mitochondrial function, promotes ROS generation, and induces apoptosis. These actions lead to the inhibition of cell migration and colony formation, underscoring ALD’s multifaceted cytotoxic impact, including apoptosis induction, growth inhibition, and mitochondrial dysfunction. The bioactive components of ALD, such as Atractylenolide II and III (42), demonstrate strong molecular interactions with the androgen receptor (AR) as shown by molecular docking studies (43). Since AR signaling plays a crucial role in prostate cancer progression, these interactions suggest that ALD may effectively disrupt AR-driven pathways, thereby inhibiting PC3 and DU145 cell proliferation and survival (44). By simultaneously triggering ROS-mediated apoptosis and impairing cellular processes essential for metastasis (45, 46), ALD emerges as a potential multi-targeted therapeutic candidate for prostate cancer treatment. Our findings also indicate that the combined binding strength of Atractylenolide II and III may approximate or even surpass that of ENZ, highlighting the therapeutic advantage of herbal formulations like ALD, where multiple active compounds work synergistically to achieve a more stable and comprehensive interaction with target receptors. This multi-component synergy, typical of herbal medicines, may provide broader efficacy compared to single-agent therapies (**Table 1**, **Figure 2**). The examination revealed that ALD exerts a significant influence on the survival of prostate cancer (PCa) cells, particularly within the PC3 and DU145 cell lines (47), as evidenced by the results obtained from the EZ-Cytotoxic kit assays. Notably, these assays demonstrated that the cytotoxic effects of ALD are both time- and dose-dependent, indicating that prolonged exposure and higher concentrations of ALD correlate with increased inhibition of cancer cell proliferation. This observed cytotoxicity underscores ALD’s capacity to directly impede the growth of cancer cells while sparing normal cells, thereby suggesting a cancer-specific effect of ALD. Further validation of the decrease in cell viability was achieved through colony formation assays, which demonstrated a marked reduction in the ability of PCa cells to form colonies following ALD treatment. This reduction in colony formation not only indicates the elimination of cancer cells but also highlights ALD’s ability to hinder their capacity for proliferation and the establishment of new tumor colonies. These findings underscore the potential of ALD as a promising anti-cancer agent. Overall, the results are compelling, suggesting that ALD may not only eradicate existing cancer cells but also play a crucial role in preventing the formation of new tumors, thereby positioning it as a valuable candidate for further investigation in cancer therapeutics (**Figures 3**, **4**).

### Mechanism of apoptosis and ROS generation

One of the significant discoveries in this study is ALD’s ability to induce apoptosis in PCa cells through mitochondrial dysfunction and ROS generation. ALD’s impact on mitochondrial membrane potential (MMP) suggests an intrinsic pathway of apoptosis, where mitochondrial instability leads to the release of pro-apoptotic factors like cytochrome C (48). This finding aligns with current research suggesting that MMP disruption and elevated Ca^2+^ levels are critical factors in apoptosis induction by anticancer agents (49). The JC-1 assay results confirmed ALD-induced MMP depolarization, and the increase in intracellular Ca^2+^ further supports its role in apoptosis initiation (**Figure 5**). Additionally, ALD-induced ROS elevation in PCa cells reinforces its pro-apoptotic activity. ROS, highly reactive molecules that cause oxidative stress, have been linked to cell death in various cancer studies. The observed increase in ROS production suggests that oxidative stress is a key mechanism underlying ALD’s cytotoxicity (**Figure 6**). The involvement of ROS in facilitating apoptosis has been extensively studied, and the elevation of ROS production by ALD provides additional evidence for its pro-apoptotic properties (50). Our study further demonstrated that ALD treatment results in the release of cytochrome C, a critical step in mitochondrial-mediated apoptosis, which triggers downstream apoptotic pathways. The increased ATF4 levels in ALD-treated cells suggest endoplasmic reticulum stress and support the involvement of mitochondrial pathways in apoptosis (**Figure 7**).

### Suppression of EMT and potential anti-metastatic effects

The epithelial-to-mesenchymal transition (EMT) is crucial in cancer metastasis, where epithelial cells transform, gaining motility and invasive potential (51, 52). Our findings indicate that ALD suppresses EMT in PC3 and DU145 cells by modulating the TGF-β signaling pathway (**Figure 8**). ALD treatment significantly downregulated TGF-β and N-cadherin while upregulating E-cadherin, signaling a reversal of EMT (53) and suggesting reduced metastatic potential. TGF-β inhibition by ALD indicates that it may hinder metastasis, as TGF-β is a known regulator of EMT (54). Future studies are warranted to clarify ALD’s interactions with TGF-β pathway components and the molecular basis of its anti-EMT effects.

### Therapeutic implications and multi-targeted approach

The results of this study indicate that ALD has the potential to act as a multi-targeted treatment for prostate cancer by inducing programmed cell death, increasing ROS, altering mitochondrial function, and inhibiting EMT. Such a multi-targeted approach is highly advantageous in cancer therapy as it reduces the likelihood of cancer cells developing resistance. Moreover, being a natural compound, ALD may offer lower toxicity and fewer side effects than conventional chemotherapies, potentially providing a safer alternative

### Limitations and future directions

While this study provides strong evidence of ALD’s anticancer potential, further research, including *in vivo* xenograft experiments and testing on primary human prostate cancer cells, is essential to strengthen the evidence of ALD’s efficacy in cancer treatment. Such models would help validate these findings in a more physiological context and are crucial for translating these results into clinical applications. Future studies should investigate the detailed molecular pathways through which ALD affects prostate cancer cells, focusing on the complex mechanisms of mitochondrial dysfunction and ROS generation. Additionally, conducting clinical trials alongside animal studies, particularly xenograft models, would provide a more comprehensive understanding of ALD’s efficacy and safety. Exploring the potential synergy of ALD with other anticancer agents could also lead to innovative combination therapies. Further research should assess the long-term effects and potential side effects of ALD treatment, ensuring its safety and effectiveness for therapeutic use. Collectively, this study highlights ALD’s promising role as a multi-targeted therapeutic agent against prostate cancer, laying a solid foundation for future research into its clinical applications.

## Conclusion

**Figure 9:**
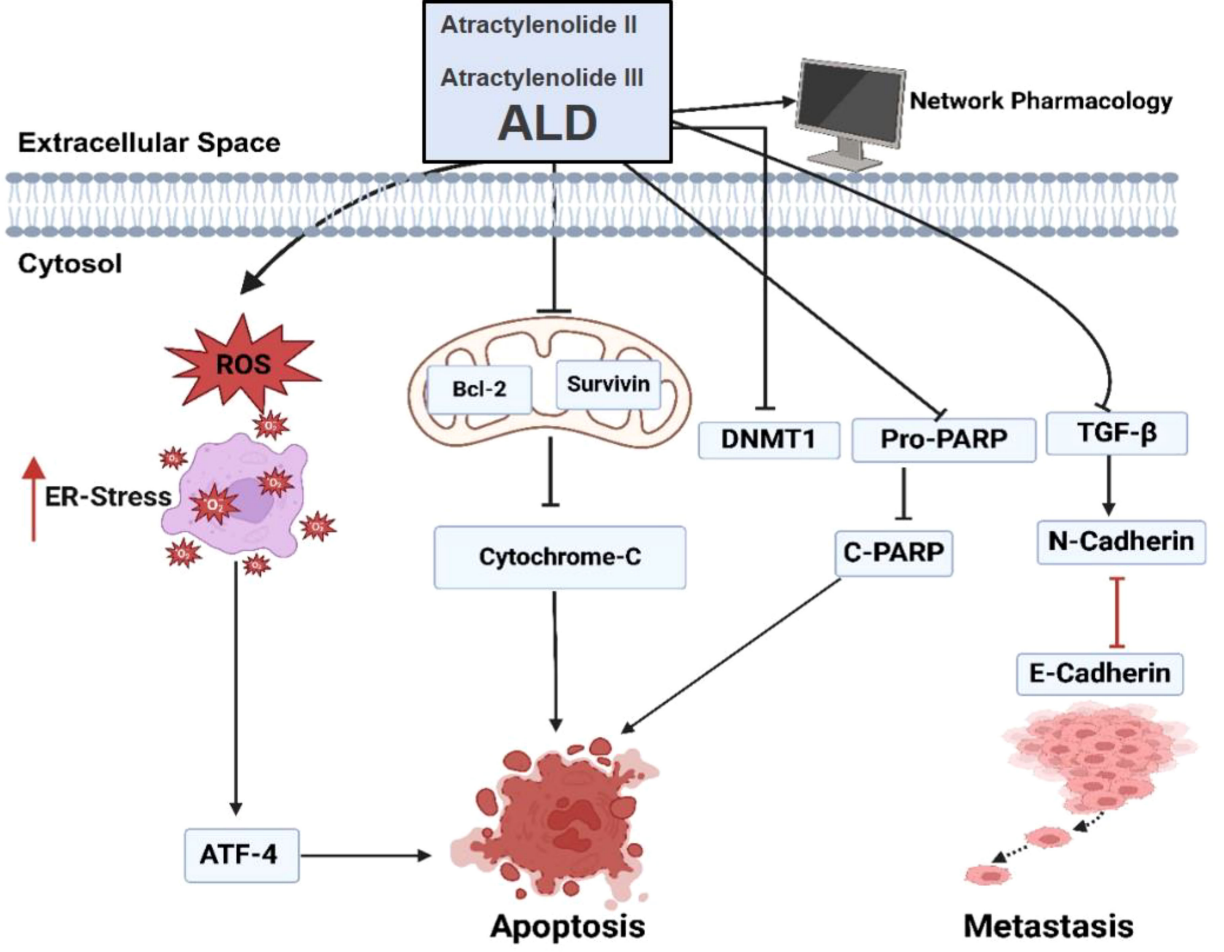
ALD-induced apoptosis and inhibition of EMT in PC3 and DU145 cells. Treatment with ALD (containing Atractylenolide II and Atractylenolide III) reduces mitochondrial membrane potential, leading to cytochrome c release and activation of apoptosis markers, including cleaved PARP, ATF4, and E-cadherin. Concurrently, ALD suppresses levels of pro-survival proteins (pro-PARP, survivin, and Bcl-2) and EMT-related proteins (DNMT1 and N-cadherin). These molecular changes suggest that ALD inhibits EMT progression, thereby promoting apoptosis in prostate cancer cells.

In summary, this study highlights the significant anticancer properties of *Atractylodes lancea* DC. (ALD) in targeting prostate cancer. ALD exhibits cytotoxic effects, induces apoptosis through ROS generation and mitochondrial disruption, and inhibits epithelial-to-mesenchymal transition (EMT) by modulating TGF-β signaling pathways (**Figure 9**). These findings suggest that ALD could be a promising therapeutic agent, offering a multifaceted approach to combat prostate cancer. However, further research, including clinical trials, is essential to validate and expand upon these therapeutic effects in clinical settings.

## Data Availability

The raw data supporting the conclusions of this article will be made available by the authors, without undue reservation.
